# Complications after pediatric percutaneous endoscopic gastrostomy: comparison of the push and pull technique

**DOI:** 10.1136/wjps-2023-000687

**Published:** 2024-01-28

**Authors:** Mona Takalo, Tarja Iber, Reija Autio, Topi Luoto

**Affiliations:** 1 Faculty of Medicine and Health Technology, Tampere University, Tampere, Finland; 2 Department of Pediatric Surgery, Tampere University Hospital, Tampere, Finland; 3 Faculty of Social Sciences, Tampere University, Tampere, Finland

**Keywords:** Gastroenterology

## Abstract

**Purpose:**

Various complications are associated with percutaneous endoscopic gastrostomy (PEG) procedures in children. The push technique is being increasingly used, but its complications are insufficiently characterized. We aimed to assess all complications related to PEG procedures and compare the safety of the pull and push techniques.

**Methods:**

Retrospective review of consecutive pediatric patients who underwent PEG between 2002 and 2020.

**Results:**

In total, 216 children underwent 217 PEG procedures. The push technique was used in 138 (64%) cases, and the pull technique in 79 (36%) cases. The median follow-up time was 6.1 (0.1–18.3) years. The complication rate was high (57%) and patients experienced complications years after the procedure. Overall, 51% and 67% of patients experienced complications in the push and pull groups, respectively. The rates of minor and major complications were higher in the pull group than in the push group (63% vs 48%, *p*=0.028; and 11% vs 6%, *p*=0.140, respectively). Reoperation was also more common in the pull group (17% vs 7%, *p*=0.020).

**Conclusions:**

The overall complication rate of PEG procedures is high. Fortunately, most complications are mild and do not require reoperations. The increasing push technique appears to be safer than the traditional pull technique. Significant long-term morbidity is related to gastrostomies in children.

**WHAT IS ALREADY KNOWN ON THIS TOPIC**

- Various complications are related to percutaneous endoscopic gastrostomy procedures in children.
- The push technique is increasingly used and replacing the pull technique, but the rate and type of complications associated with the push technique remain scarcely reported.

**WHAT THIS STUDY ADDS**

- The rate of complications after pediatric percutaneous endoscopic gastrostomy is higher than expected, and a significant proportion of patients experience multiple minor complications in the long term.
- The rate of complications is lower with the push technique than with the traditional pull technique, although T-fastener-related complications are common.

**HOW THIS STUDY MIGHT AFFECT RESEARCH, PRACTICE OR POLICY**

- The increasing push technique seems to be safer than the traditional pull technique and is the preferable method for placing percutaneous endoscopic gastrostomy in children.
- Caregivers should be informed of the potential long-term morbidity of percutaneous endoscopic gastrostomy procedures.

## Introduction

In the last few decades, percutaneous endoscopic gastrostomy (PEG) has become one of the most common pediatric procedures performed.^1^ Various underlying diseases compromise the oral intake of nutrients and medications, requiring the use of nasogastric or nasojejunal tubes. PEG procedure and nutritional support via gastrostomy are indicated in children requiring prolonged enteral tube feeding.^2–5^

Operative techniques have evolved since 1980, when Gauderer *et al* introduced the first PEG.^6^ The traditional pull technique has been accompanied by a push, or in other words, an introducer or one-step technique, and also laparoscopy-assisted and radiologically inserted gastrostomies have been introduced. The push technique was first described in 1984 and is increasingly being used in children.^7^ The main advantage over the traditional pull technique is that a second general anesthesia for tube removal and replacement with a low-profile device may be avoided. Despite the widespread use of the push technique, the rate and profile of postoperative complications, and long-term outcome remain sparsely characterized. Recent studies have highlighted the safety of this one-step technique.^8–10^

In children, the overall PEG complication rate varies between 21% and even 78%, depending on the follow-up time and how the complications are described.^8 10–14^ Outcomes from both the pull and push technique operations have been compared, and it seems that in children, the newer push technique is at least as safe as the traditional technique.^13 15^ Complications seem to be common, but fortunately, minor complications form the notable majority, and major complications occur in only 10–13% of cases.^12 16 17^

A twofold aim of this study was to report all short-term and long-term complications related to PEG procedures in our single-center study and to compare the safety of the pull and push PEG techniques.

## Patients and methods

### Study population

The medical records of all consecutive patients aged under 18 years who underwent PEG between January 2002 and July 2020 at the Tampere University Hospital were reviewed. A total of 216 patients were included in this study. One patient underwent two separate PEG procedures and was considered twice for the analysis. Altogether, 217 procedures were used to establish our database. The data were collected between July and November 2020.

### Study design

After ethical approval, medical records were retrospectively reviewed and the following variables were collected: date of birth, sex, underlying diseases, previous surgical procedures, age at the time of surgery, preoperative weight, indication of the operation, surgical technique, and postoperative complications. Postoperative complications were classified according to the European Society for Paediatric Gastroenterology Hepatology and Nutrition Position Paper^3^ as minor or major and early or late. Overgranulation was considered as a complication if the granulation tissue was treated with silver nitrate or excised in the operating room. Site infections were considered as complications if they were treated with oral antibiotics. If intravenous antibiotic treatment was needed, the infection was considered cellulitis.

### Patient management

Patients with malnutrition and prolonged need for nasogastric tube application were referred to our department. Gastrostomy was performed under general anesthesia. All the patients received a single dose of antibiotic prophylaxis with cefuroxime (20 mg/kg) prior to the procedure. Postoperative nutrition was initiated within 12–24 hours after surgery. Patients were discharged when full enteral nutrition was achieved, typically 1–3 days after the procedure. Until 2009, the pull technique was primarily used. After 2010, the push technique became the primary method for gastrostomy applications due to advantage of avoiding second general anesthesia. All patients were postoperatively followed up by a specialist nurse. In patients who underwent PEG with the push technique, the T-fastener sutures were cut by our nurse after 3 weeks if they had not fallen off due to absorption. After 3 months, the gastrostomy button was changed for the first time by a specialist nurse. Patients were instructed to contact the specialist nurse regarding all postoperative issues. A pediatric surgeon was consulted if needed. This follow-up protocol has been used throughout the study period.

### Operative techniques

The pull technique was performed as previously described by Gauderer *et al*.^6^ The MIC PEG kit was used in the primary procedure and the MIC-KEY button (Avanos Medical) in the replacement. In the push technique, a gastrostomy button was placed using the MIC-KEY Introducer Kit (Avanos Medical). Upper gastrointestinal endoscopy was carried out, and the stomach was insufflated and transilluminated. Gastropexy was performed using three T-fasteners inserted in triangular configuration under endoscopic control. A channel for the gastrostomy button was created in the center of the T-fasteners using the Seldinger technique and serial 18 Fr dilatator. The length of the gastrostomy button was measured. Finally, a 14 Fr gastrostomy button was inserted, and the balloon was filled with 5 mL of sterile water. Laparoscopy was not used in either technique.^4^

### Statistical analyses

The collected data formed our database and were analyzed using IBM SPSS Statistics V.27 software. Categorical variables are presented as counts and percentages, whereas continuous variables are presented as medians and ranges. Categorical independent variables were compared using the Χ^2^ test, and continuous variables were compared using the Mann-Whitney U test. Kaplan-Meier curves and log-rank tests were used to analyze the occurrence and timing of postoperative complications. There were no missing data. The level of statistical significance was set at p<0.05.

### Patient and public involvement

The medical records of the study population were collected retrospectively, and consequently involving other healthcare workers alongside the research team was not necessary.

## Results

### Patient characteristics

Overall, 216 children underwent 217 PEG procedures at a median age of 1.7 (0.2–17.8) years. Push and pull techniques were used in 138 (64%) and 79 (36%) cases, respectively. Patient characteristics and comparisons of the surgical techniques are summarized in table 1. The push and pull technique groups were comparable in terms of sex, age, indications, previous abdominal surgery, and preoperative weight. In the push technique group, the median operating time was 25 min (range 10–67, *n*=83). Overall, the median follow-up time was 6.1 (0.1–18.3) years, and there was a statistically significant difference between the groups.

**Table 1:** Characteristics of patients who had undergone percutaneous endoscopic gastrostomy and comparisons between push and pull techniques

	All (*n*=217)	Push (*n*=138)	Pull (*n*=79)	*P* value
Male	117 (53.9%)	79 (57.2%)	38 (48.1%)	0.193
Age (years)	1.7 (0.2–17.8)	1.5 (0.2–17.3)	1.7 (0.3–17.8)	0.363
Indications				
Neurological condition	111 (51.2%)	75 (54.3%)	36 (45.6%)	0.218
Oncological disease	27 (12.4%)	19 (13.8%)	8 (10.1%)	0.428
Cardiac disease	22 (10.1%)	12 (8.7%)	10 (12.7%)	0.349
Gastrointestinal	16 (7.4%)	9 (6.5%)	7 (8.9%)	0.516
Other	41 (18.9%)	23 (16.7%)	18 (22.8%)	0.271
Previous abdominal surgery	36 (16.6%)	24 (17.4%)	12 (15.2%)	0.675
Preoperative weight (kg)	9.5 (3.5–74.0)	9.4 (3.5–41.6)	10.5 (4.5–74.0)	0.517
Follow-up time (years)	6.1 (0.1–18.3)	4.3 (0.1–10.5)	12 (0.8–18.3)	0.001

Number of patients with percentages for categorical variables and medians (ranges) for continuous variables.

The indications for PEG were divided into five main groups. Neurological disorders comprised the largest group and included neurodevelopmental disorders, neuromuscular disorders, and neonatal or traumatic anoxic brain damage. Oncological conditions included brain tumors, renal tumors, malignant hematological diseases, and sarcomas. Gastrointestinal disorders included esophageal atresia, Hirschsprung’s disease, short bowel syndrome, and gastroschisis. The remaining conditions were categorized as other and included cystic fibrosis, laryngomalacia, palatoschisis, IgG deficiency, Langerhans histiocytosis, tyrosinemia, and various kidney diseases.

### Complications

Overall, 123 (57%) patients experienced 198 postoperative complications. Fifty-nine patients (27% of all patients and 48% of patients with complications) had more than one complication. Complications occurred early (within 30 days postoperatively) in 53 patients (43%) and late (later than 30 days postoperatively) in 70 patients (57%). The overall complication rate was higher in the pull technique group (67%) than in the push technique group (51%; *p*=0.019). The rate of postoperative complications in patients who had undergone previous abdominal surgery was comparable with the patients who had not undergone previous abdominal surgery (56% vs 57%, *p*=0.881).

### Major complications

**Figure 1:**
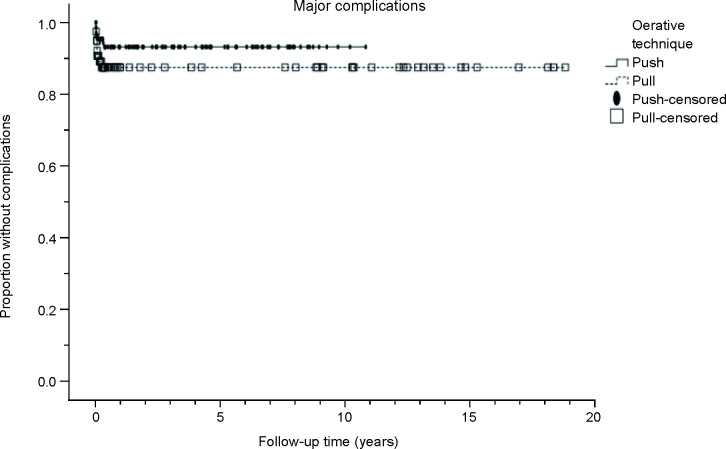
Cumulative survival (Kaplan-Meier curve) without major complications in different operative technique groups during the follow-up period (years).

Major complications are presented in table 2. Overall, 8% of the patients experienced major complications. The rate was higher in the pull technique group (11%) than in the push technique group (6%); however, the difference was not statistically significant (*p*=0.140). No mortality was associated with the PEG procedures. Figure 1 shows that the cumulative survival rates without major complications with the push versus pull technique at 30 days, 6 months, 1 year, and 3 years were 96% vs 91%, 94% vs 89%, 94% vs 89%, and 94% vs 89%, respectively. A comparison of cumulative survival without major complications between the operative techniques showed no statistically significant difference (*p*=0.167).

**Table 2:** Comparison of complications between patients who had undergone push and pull technique percutaneous endoscopic gastrostomy

	All (*n*=217)	Push (*n*=138)	Pull (*n*=79)	*P* value
Major	17 (7.8%)	8 (5.8%)	9 (11.4%)	0.140
Abscess	3 (1.4%)	2 (1.4%)	1 (1.3%)	0.911
Buried bumper syndrome	3 (1.4%)	0 (0.0%)	3 (3.8%)	0.021
Cellulitis	3 (1.4%)	1 (0.7%)	2 (2.5%)	0.273
Gastrocolic fistula*	2 (0.9%)	1 (0.7%)	1 (1.3%)	0.688
Peritonitis	1 (0.5%)	1 (0.7%)	0 (0.0%)	0.448
Pneumonia	4 (1.8%)	2 (1.4%)	2 (2.5%)	0.568
Septic infection	2 (0.9%)	1 (0.7%)	1 (1.3%)	0.688
Minor	116 (53.5%)	66 (47.8%)	50 (63.3%)	0.028
Overgranulation	64 (29.5%)	37 (26.8%)	27 (34.2%)	0.252
Site infections	50 (23.0%)	25 (18.1%)	25 (31.6%)	0.023
Tube degradation	4 (1.8%)	2 (1.4%)	2 (2.5%)	0.568
Unplanned removal	43 (19.8%)	24 (17.4%)	19 (24.1%)	0.236
Other†	13 (6.0%)	13 (9.4%)	0 (0.0%)	0.005
Reoperations	22 (10.1%)	9 (6.5%)	13 (16.5%)	0.020

*Includes gastrocolic and gastrointestinal fistulas.

†Includes unplanned T-fastener removals and T-fastener infections.

### Minor complications

**Figure 2:**
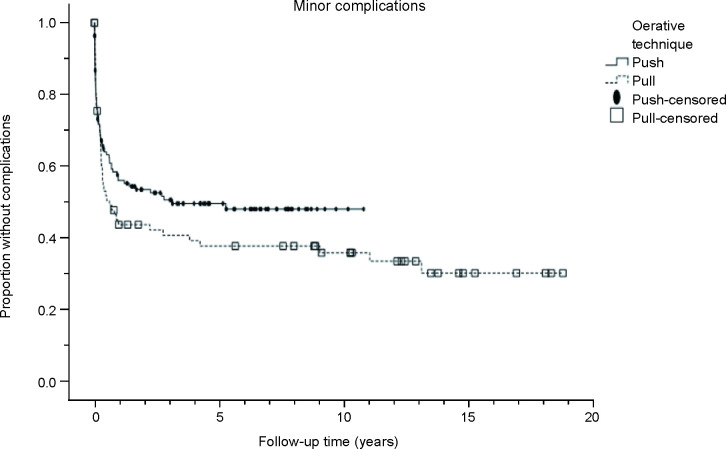
Cumulative survival (Kaplan-Meier curve) without minor complications in different operative technique groups during the follow-up period (years).

The minor complications are shown in table 2. Overall, 54% of the patients experienced minor complications. Fifty-two patients (45% of patients with minor complications) had more than one minor complication. The complication rate was significantly higher in the pull technique group (63%) than in the push technique group (48%; *p*=0.028). Minor complications were mainly caused by overgranulation, site infections, and unplanned removal, which together accounted for 87% of all minor complications and 79% of all complications in our study. The T-fasteners used in the push technique caused 11 infections requiring early removal in nine patients. The cumulative survival rates without minor complications with the push versus pull technique at 30 days, 6 months, 1 year, and 3 years were 80% vs 77%, 64% vs 52%, 58% vs 46%, and 54% vs 43%, respectively (figure 2). A comparison of cumulative survival without minor complications between the operative techniques showed no statistically significant difference (*p*=0.180).

### Reoperations

Reoperation after PEG was performed in 22 (10%) patients: 9 (7%) push techniques and 13 (17%) pull techniques, as seen in table 2. The difference between groups was statistically significant (*p*=0.020) and considering that in the pull technique group, three patients had more than one reoperation, the disparity between the groups was even greater.

With patients who underwent push PEG reoperations, reoperations were done due to unplanned PEG button removal (*n*=5), T-fastener migration to the skin (*n*=2), T-fastener infection/abscess (*n*=1), and gastrointestinal fistula (*n*=1). The patient with a gastrointestinal fistula required laparotomy 4 months postoperatively to suture the small bowel fistula and reposition the gastrostomy.

After pull PEG, 13 patients required 17 reoperations due to buried bumper syndrome (*n*=3), PEG tube degradation (*n*=2), abscess/infection (*n*=2), unplanned removal (*n*=8), gastrocolic fistula (*n*=1), and gastrostomy tube functional problems (*n*=1). The patient with a gastrocolic fistula required laparotomy 7 days postoperatively to suture the transverse colon fistula and reposition the gastrostomy.

## Discussion

Short-term and long-term complications associated with pediatric PEG procedures were retrospectively analyzed in a cohort of 216 patients. Major and minor complications were compared according to the surgical technique used. The overall complication rate was notable and 27% of patients experienced several complications. The majority of complications consisted of well-known minor complications, such as overgranulation, site infections, and unplanned removal of the gastrostomy tube or button. Both push and pull techniques were associated with a significant number of complications, necessitating additional surgical procedures in 10% of patients. With the push technique, the overall rates of minor complications were lower. Also, different types of complications were almost without exception, more common in the pull group than in the push group. T-fastener-related complications were reported in 10% of the patients, which was a clear disadvantage of the push technique. A large number of patients experience complications years after the primary operation.

In children, the rate of PEG complications is extremely variable depending on the definition of complications and duration of follow-up. In a review of 4631 patients by Balogh *et al*,^12^ 33% of patients developed minor complications and 10% of patients developed major complications. In one of the largest cohort studies^15^ (*n*=450) with a 120-day follow-up time comparing seven different gastrostomy techniques, 5.3% of all patients experienced dislodgement requiring a return to the operating room, 51.5% experienced overgranulation, and 29% of patients had leakage at the stoma. The push technique was found to have the lowest rate of complications. In the largest retrospective series^8^ including 679 children who underwent PEG with the push technique, the median follow-up time was 2.8 years and rates of major, early, and late complications were <2%, 15.9%, and 78.0%, respectively. In the aforementioned studies, complications have been reported non-uniformly, making comparison with our results challenging. However, mainly our findings are consistent. Major complications, which cause a significant burden to the child and family, developed in 8% of all patients. The rate of minor complications was high, over half of the patients, which is partly related to our long follow-up period (figure 2) and careful retrospective review of patient records. The long-term morbidity after PEG was notable and is presumably independent of the used surgical technique. Although long-term complications are mainly minor and do not require surgical treatment, the burden for the family and healthcare seems to continue years after the procedure.

The push technique is increasingly used in many centers and has become the primary PEG technique in our hospital and many others. The main advantage of this technique is the need for only one general anesthesia.^4 11^ Many patients have considerable risk factors for anesthesia, and multiple operations increase the burden of the entire family. Despite its widespread use, the literature regarding the safety of this technique has been limited. New cohort studies have recently been published to support this trend.^8 10^ Comparative studies on different PEG techniques do not uniformly show that the push technique is superior to other techniques; however, none of the studies have shown it to be inferior in children when analyzed for safety.^9 11 13^ When comparing the complications of all different gastrostomy techniques (including laparoscopy and radiographically assisted) in children, the optimal procedure remains controversial.^18 19^ In recent meta-analyses,^18 19^ laparoscopic technique had significantly fewer major complications compared with PEG, which is probably due to improved visualization of the abdominal cavity as they both discussed. Then, PEG techniques are less invasive, the operating time is shorter, and the procedure is technically less demanding, Major complications may be avoided with an uncompromising surgical technique (adequate insufflation of the stomach, transillumination and perpendicular insertion of the T-fasteners and guide wire) and using laparoscopy when in doubt of safety. Regarding percutaneous endoscopic techniques, the push technique seems to achieve its role as the primary technique. Our results provide an additional justification for support. As discussed by Dahlseng *et al*,^10^ the push technique is safe, and according to our observations, it takes only 15 min to be performed by experienced pediatric surgeons. The rate of T-fastener-related complications is significant and clearly a disadvantage of the technique.^8 10 20^ We share the same clinical experience that optimal tightness and duration of the T-fasteners seem to decrease the complications.^10^ In our department, the percutaneous and endoscopic push technique remains the primary method for gastrostomy placement, and laparoscopy is reserved for cases in which safety of this method is in doubt during the procedure.

Our cohort was relatively large and heterogeneous, including all consecutive children who had undergone PEG for various indications. None of the patients were excluded; therefore, the results can be generalized. The long duration of follow-up, centralized postoperative care, and systematic review of patient records ensured the coverage of all clinically significant complications. However, the retrospective nature of the study may have underestimated the rate of complications, particularly minor complications. From the year 2002 onward, data were reliably collected from electronic medical records and thus all patients between 2002 and 2020 were included and power calculations were not done. Patients who underwent the pull PEG had a longer follow-up period because the pull technique was the primary operative technique used until 2010. Then again, in the patients who underwent push PEG, the follow-up time was relatively long and included all clinically significant postoperative complications. During the long study period and follow-up, the treatment and medical devices, and the aftercare of minor complications may not have been completely uniform and several surgeons have been performing PEG operations. The possible influence of these confounders on the study could not be taken into account.

In summary, pediatric PEG procedures are associated with a significant number of complications that require hospital resources to address. The majority of complications are mild and develop over a long period of time. The increasing push technique seems to be safer than the traditional pull technique, although T-fastener-related complications are common.

## Data Availability

Data may be obtained from a third party and are not publicly available. Data were collected from the medical records of Tampere University Hospital.
